# Failure Behavior of Granite Affected by Confinement and Water Pressure and Its Influence on the Seepage Behavior by Laboratory Experiments

**DOI:** 10.3390/ma10070798

**Published:** 2017-07-14

**Authors:** Cheng Cheng, Xiao Li, Shouding Li, Bo Zheng

**Affiliations:** 1 Key Laboratory of Shale Gas and Geoengineering, Institute of Geology and Geophysics, Chinese Academy of Sciences, Beijing 100029, China; cheng@mail.iggcas.ac.cn (C.C.); lsdlyh@mail.iggcas.ac.cn (S.L.); zhengbo@mail.iggcas.ac.cn (B.Z.); 2 College of Earth Sciences, University of Chinese Academy of Sciences, Beijing 100049, China

**Keywords:** granite material, failure behavior, seepage behavior, confinement, water pressure

## Abstract

Failure behavior of granite material is paramount for host rock stability of geological repositories for high-level waste (HLW) disposal. Failure behavior also affects the seepage behavior related to transportation of radionuclide. Few of the published studies gave a consistent analysis on how confinement and water pressure affect the failure behavior, which in turn influences the seepage behavior of the rock during the damage process. Based on a series of laboratory experiments on NRG01 granite samples cored from Alxa area, a candidate area for China’s HLW disposal, this paper presents some detailed observations and analyses for a better understanding on the failure mechanism and seepage behavior of the samples under different confinements and water pressure. The main findings of this study are as follows: (1) Strength reduction properties were found for the granite under water pressure. Besides, the complete axial stress–strain curves show more obvious yielding process in the pre-peak region and a more gradual stress drop in the post-peak region; (2) Shear fracturing pattern is more likely to form in the granite samples with the effect of water pressure, even under much lower confinements, than the predictions from the conventional triaxial compressive results; (3) Four stages of inflow rate curves are divided and the seepage behaviors are found to depend on the failure behavior affected by the confinement and water pressure.

## 1. Introduction

Granite is usually considered as a material with high strength, low permeability, and good excavation stability, etc., so that it is always applied as one of the host rocks for the geological repositories for High Level Waste (HLW) disposal [1,2]. As a natural barrier to prevent the transportation of radionuclide, the failure properties and seepage characteristics of granite are of great importance [3,4,5,6,7,8,9]. Actually, these characteristics are also paramount for many other types of rock engineering work such as mining, tunneling, and some engineering geological problems like dam failures, landslides, and injection induced earthquakes [10,11].

In recent decades, extensive studies on the mechanical or seepage behaviors of rock or rock mass have been carried out examining the effects of confinement or/and water. The key research is as follows:

(1) Effect of water content on the mechanical behavior of rock. Numerous experimental studies have been conducted to study the effect of moisture content on the mechanical behaviors of different types of rock including sandstone, limestone, shale, siltstone, gypsum, chalk, granite, tuff, andesite, etc. [12,13,14,15,16,17,18,19,20,21]. Reduction in strength, or Young’s modulus, with different degrees were found for most of the rock types, with certain contents of water due to different reasons such as mineral deterioration, pore pressure increase induced reduction of effective stress, capillary tension decrease, decrease of fracture energy or friction coefficient, etc. [21]. It was observed that strength reduction is more significant for clay-rich rock than quartz-rich rock [19]. Hashiba and Fukui’s experiments demonstrated that the reduction rates of uniaxial compression strength and uniaxial tension strength for granite are not as high as several other rock types [20].
(2) Effects of confinement and water pressure on the mechanical behavior of rock. Based on linear elastic fracture mechanics (LEFM), some authors gave the theoretical equations for identifying the initiation, propagation of cracks affected by various stress conditions and pore water pressures [22,23,24,25]. Different numerical tools were also applied to give parametrical studies on the pore water pressure induced fracturing [11,26]. In laboratory experiments, Vogelhuber et al. (2004) investigated the deformation behavior of kakirite considering pore water pressures under triaxial conditions [27]. Rong et al. (2013) studied the stress thresholds including crack initiation stress *σ*_ci_, crack damage stress *σ*_cd_, and peak stress *σ*_f_ of sandstone affected by confinement and water pressure [28]. Yang et al. (2014) reported a consistent study on the short-term strength, deformation properties and creep strain behaviors of red sandstone considering the influences of confinement and water pressure [29]. In petroleum engineering, the effect of stress condition and pore water pressure is usually considered for studying hydraulic fracturing properties [7,30,31].
(3) Effect of stress condition on seepage characteristics of rock mass or rock discontinuities. The different stress conditions may lead to the fracture opening/closure or shear dilation, which is sensitive to the seepage characteristics. Laboratory experiments under triaxial compression or direct shear have been carried out to study the relationship between the confinement or normal stress and permeability on rock mass or a single rock fracture [10,12,32,33,34]. Besides, various models of permeability tensors were built to describe the permeability of fracture network in the rock mass and have been used in field engineering under different stress conditions [35,36,37].
(4) Effects of confinement and water pressure on evolutionary seepage characteristics of rock during the process of damage. Different seepage characteristics were observed in different parts in the excavation damaged zone (EDZ) in the Underground Rock Laboratory (URL) in Canada [38] and the Mont Terri Rock Laboratory in Switzerland [39]. It was revealed that the surrounding rock may exhibit different seepage behavior during the damage process under different stress conditions and water pressures. Many laboratory experiments have been conducted to study the permeability evolution of rock samples including limestone, sandstone, granite, etc. under triaxial compression with water pressure [40,41,42,43,44,45,46,47]. Some fitted empirical permeability evolution models were built to describe the seepage behaviors based on the laboratory tested permeability values under different confinements and water pressures [41,47]. Some other laboratory studies analyzed the relationship between the permeability evolution and the cracking process based on the direct method of crack statistics in the cutting planes after the experiments [42] and indirect methods of ultrasonic wave velocity or deformation monitoring [48,49].

Based on the literature reviews listed above, it has been well recognized that the initially intact rock will be damaged and fractured under non-hydrostatic conditions affected by different confinements and water pressures. In addition, the failure process will have influences on the evolutionary seepage behavior of the rock. Nonetheless, there are several problems requiring more studies: (1) In addition to the strength and deformation behaviors, the fracturing patterns of rock should also be studied under the effects of confinement and water pressure, and thereafter, the seepage behaviors should be examined in combination with these failure behaviors; (2) The relationship between the damage process and seepage characteristics are investigated. However, the influence of water pressure on the damage process is always ignored. What is more, the damage behavior is always measured by indirect or statistical methods, while the real fracturing modes cannot be described; (3) Empirical models of permeability considering confinement and water pressure are built, while the mechanism should be better understood.

As we know, different parts of the host rock after excavation may be under different stress and water conditions. Aiming at obtaining a better understanding on the failure mechanism of the host rock under different confinements and water pressures, and in turn, its influence on the seepage characteristics, granite samples cored from the NRG01 borehole in Alxa area, a candidate area for China’s HLW disposal, are used for the laboratory hydro-mechanical studies under non-hydrostatic conditions in this paper. Basic properties of NRG01 granite samples and the experimental methodology will be introduced in Section 2. The influence of confinement and water pressure on the failure behaviors of NRG01 granite samples will be studied in Section 3. This will include the strength properties, stress–strain relationships and especially the fracturing patterns which are important for both mechanical stability and seepage behavior. Thereafter, the effects of the failure behaviors on seepage characteristics of NRG01 granite samples will be presented in Section 4. Finally, some discussions and conclusions will be given in Section 5.

## 2. Samples, Experimental Setup and Methods

### 2.1. Samples

The granite samples are cored from NRG01 borehole, one of the four boreholes in Alxa area for China’s site selection for HLW disposal. NRG01 borehole is located in Nuorigong sub-area where the near surface rock mass is very intact. The RQD analyses show that NRG01 drilling cores have the best rock mass quality among the four boreholes with the depth of 600 m. Consequently, extensive experimental studies have been carried out on the NRG01 granite samples, including the influence of temperature, dynamic effect, etc. [50].

**Figure 1:**
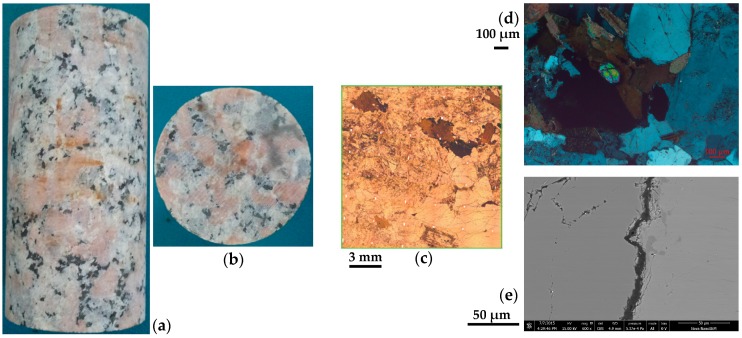
(**a**,**b**) Typical NRG01 granite samples (height: 100 mm; diameter: 50 mm) [50] and (**c**–**e**) observations of micro-cracks in NRG01 granite samples [51] (with copyright permission from Elsevier).

NRG01 granite samples, with an average density of 2.65 g/cm^3^, are pink in color and generally have coarse texture (Figure 1a,b). The granite samples have about 35% K-feldspar (grain size 2.0–22.0 mm), about 35% plagioclase (grain size 2.0–5.0 mm), about 25% quartz (grain size 2.0–4.0 mm) and about 5% other dark minerals [50]. The samples are basically intact, however, many micro-cracks with the width of about 3.0–10.0 μm can be observed in the samples based on Figure 1c–e [51].

**Figure 2:**
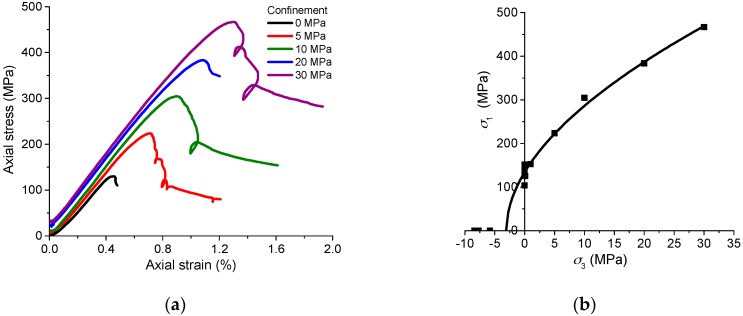
(**a**) Axial stress-stress curves of NRG01 granite samples (*σ*_3_ = 0 to 30 MPa) and (**b**) Hoek–Brown fitted envelopes based on the test results of NRG01 samples under different confinements.

The NRG01 granite samples were well prepared and placed in the air under room temperature for a few weeks before the tests. Based on the conventional uniaxial and triaxial compressive tests, NRG01 granite samples have an average UCS of 134.4 MPa, and Young’s modulus of 39.5 GPa. The cohesion and internal friction angle are 20.1 MPa and 57.5°, respectively. Brittle failure with vertical fractures and spalling can be observed in the samples under lower confinements (*σ*_3_ ≤ 10 MPa). With increasing confinements (*σ*_3_ = 20 and 30 MPa), the sample failure changes to be dominated by shear fracturing with the increasing residual strength. Figure 2 presents the typical axial stress–strain curves and peak strengths of NRG01 samples under different confinements. In addition, uniaxial compression experiments on the granite samples soaked in the water for 24 h show no obvious weakening effect on the strength or deformation behavior. The tests on heat-treated samples (up to 200 °C) exhibit very limited differences on the mechanical behaviors compared with the tests under room temperature [50]. This implies that the moisture content does not have a significant effect on mechanical properties of NRG01 granite. This could be mainly explained by the composition of hard minerals which are not sensitive to the presence of water.

### 2.2. Experimental Setup and Procedures

**Figure 3:**
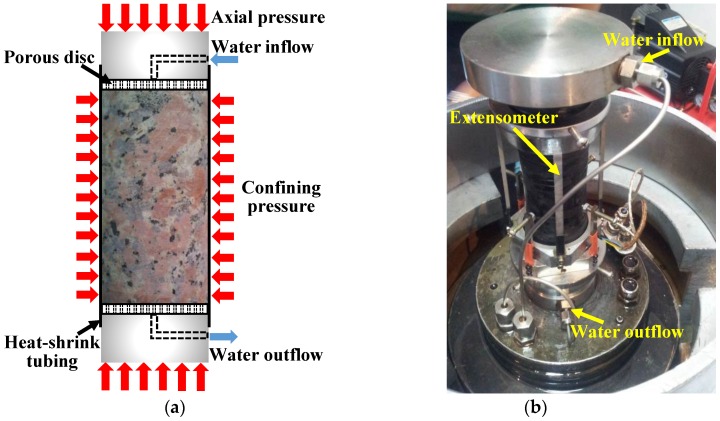
(**a**) Sketch of the experimental method; (**b**) Setup for the hydro-mechanical experiment.

This study is based on a series of hydro-mechanical (H-M) experiments carried out with TAW-2000 servo-controlled testing system in Key Laboratory of Shale Gas and Geoengineering, Chinese Academy of Sciences. For each experiment, the cylindrical granite sample (height: 100 mm; diameter: 50 mm) with the same preparing method is axially loaded under the target confinement and water pressure in the triaxial loading cell. Before the test, a pair of steel plates with water paths are attached to the two ends of the cylindrical specimen. In addition, a pair of porous steel discs are placed between the plates and the sample ends to supply a uniformly distributed water pressure to the sample. Thereafter, the combination of the specimen, porous discs and plates is packed with a heat-shrink tubing and adhesive tapes for waterproofing. When this combination is positioned in the triaxial loading cell, two steel tubes are connected to the upper and lower plates for water inflow and outflow. Additionally, an extensometer is also installed for measuring the axial and lateral strains during the process of each experiment. The sketch of the test method and the experimental setup in the triaxial loading cell are shown in Figure 3.

After the placement of the sample, the test can start according to the procedures as follows:

(1) Similar to the conventional triaxial compressive test, the triaxial cell is filled with the hydraulic fluid (oil) and increase the confining pressure to the target value.
(2) Water is injected through the upper plate and porous disc to the upper end of the specimen with a certain pressurizing rate (say, 0.1 MPa/s) to the target water pressure and keep it constant. It should be noted that the water pressure cannot be higher than the confining pressure to make sure that the heat-shrink tubing is not broken by the water pressure and the specimen is isolated from the hydraulic fluid (oil) in the triaxial loading cell.
(3) The axial load is increased with an axial strain rate of 1.0 × 10^−5^ s^−1^ until the total failure of the specimen or the occurrence of residual strength. The water inflow, water outflow, axial stress, axial and lateral strains are monitored during the process.
(4) After finishing the test, the specimen is taken out and the failure characteristics are observed.

With this method, the failure and seepage behavior of the samples can be studied during the continuous experimental process under different confinements and water pressures. It is noted that the excavation damaged zone (EDZ) is always formed in a limited area around the opening where the radial stress (confinement) is low [9]. The field pneumatic tests in the surrounding rock mass of Opalinus clay in Mont Terri Rock Laboratory show that the permeability drops to a much lower steady value within 30–40 cm (lateral part) or 50–70 cm (top part) from the opening surface [39]. In addition, the field pulse tests in Underground Rock Laboratory (URL) in Canada also show that the transmissivity decreases significantly to a stable state in the first 1 m from the granite tunnel wall [38]. It appears that the seepage behavior is more sensitive in the limited range, with more serious damage and lower confinement around the excavation. Consequently, the failure and seepage behaviors of the surrounding rock mass close to the excavation are the focus of this work, and therefore, a limited range of the confining pressures (*σ*_3_ ≤ 10.0 MPa) are considered in this study.

## 3. Failure Behavior of NRG01 Granite Samples Affected by Confinement and Water Pressure

Two groups of laboratory experiments are conducted for a better understanding of the failure behavior of NRG01 granite samples affected by confinement and water pressure. Firstly, the tests are carried out under fixed confinements (*σ*_3_ = 5.0 and 10.0 MPa) but different water pressures. Secondly, more systematic experiments are conducted under various confinements (*σ*_3_ = 1.0–10.0 MPa), and the water pressure is set as 0.8 times the corresponding confinement for each of the tests.

### 3.1. Influence of Water Pressure under Fixed Confinements

This group of tests focus on the influence of water pressure on failure behavior of NRG01 granite samples. Table 1 lists the test conditions. It should be noted here that all the samples are prepared and treated in the same way, and 0 MPa water pressure means that no water pressure is applied during the testing process for comparative studies.

**Figure 4:**
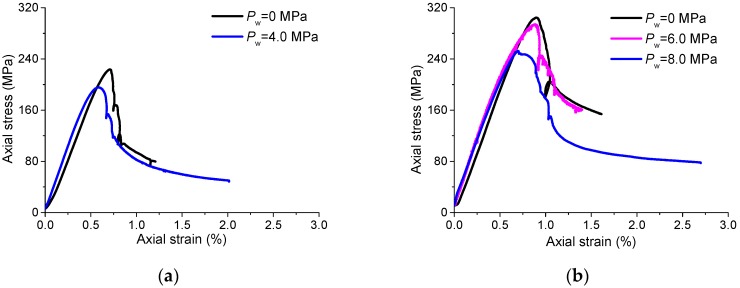
Axial stress–strain curves of the hydro-mechanical experiments affected by different water pressures. (**a**) *σ*_3_ = 5.0 MPa; (**b**) *σ*_3_ = 10.0 MPa.

**Figure 5:**
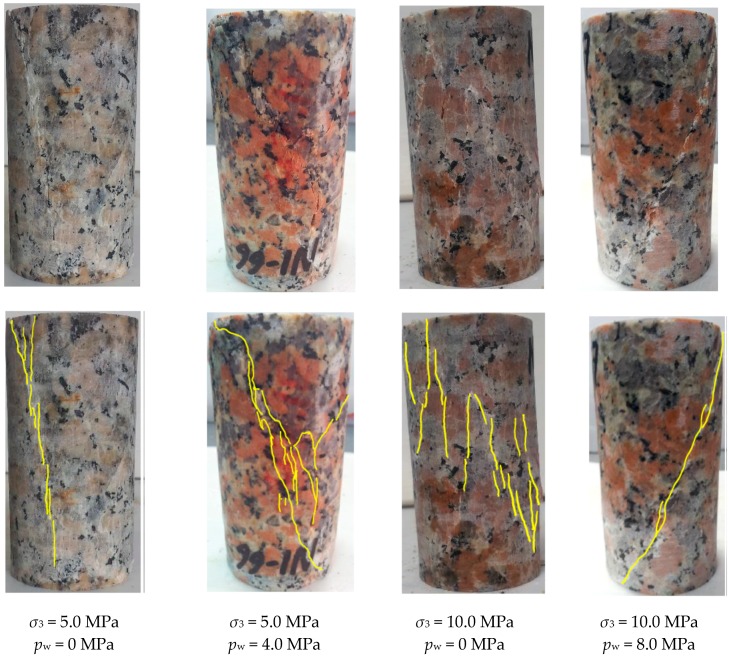
Fracturing patterns of NRG01 granite samples affected by water pressure under fixed confinements (*σ*_3_ = 5.0 MPa and 10.0 MPa). The yellow lines in the lower pictures show the fractures compared with the original pictures of the samples.

According to the obtained complete axial stress–strain curves (Figure 4) and the fracturing patterns of the specimens affected by different water pressures under fixed confinements (Figure 5), several observations can be concluded as follows:

(1) Peak strength reduction. It is obvious to find that the peak strength decreases with the increasing water pressure for each of the tests under the confinements of 5.0 and 10.0 MPa.
(2) Different fracturing patterns. It is apparent, that for the case of *σ*_3_ = 5.0 MPa, the sample under 0 MPa water pressure exhibits the failure pattern dominated by vertical tensile fractures, while the sample under 4.0 MPa water pressure shows a through shear plane mixed with some tensile cracks. For the case of *σ*_3_ = 10.0 MPa, the fracturing pattern of the specimen under 0 MPa water pressure is still dominated by echelon tensile fractures, while a shear plane goes through the specimen under 8.0 MPa water pressure. It seems that it is easier to form macro shear fractures in NRG01 granite samples under the effect of water pressure as compared to the samples under the same confinements but with no water pressure;
(3) Axial stress–strain responses. For the tests under 0 MPa water pressure, the axial stresses increase almost linearly to the peak strength and then fail in a brittle manner with sharp stress drops. However, with the increasing water pressure, there seems to be an obvious yielding process for the pre-peak stress–strain curves and the stress drop turns to be a gradual process during the post-peak period.

These observations refer to the failure behavior of NRG01 granite affected by water pressure, which should be well understood as they may play important roles in the stability as well as seepage characteristics of the host rock.

The reduction of peak strength under the effect of water pressure has also been reported in the studies on red sandstone [29]. In this study, for NRG01 granite samples whose strength properties are not sensitive to the content of moisture, we try to understand the strength weakening induced by water pressure from the following two aspects: (1) As shown in Figure 1, there are lots of pre-existing micro cracks in the apparently intact NRG01 granite samples, which supply the paths for water flow and therefore, pore water pressures will be formed in the samples. According to the analyses of Deng et al. [24] based on linear elastic fracture mechanics (LEFM) and Coulomb–Mohr criterion, it is found that both fracture toughness *K*_IC_ and *K*_IIC_ for the tips of the pre-existing micro cracks will be reduced with the increasing pore water pressures; (2) Based on the aforementioned observations from Figure 5, the cracks coalesce to form shear planes for the NRG01 granite samples under the effect of water pressure, so the Coulomb–Mohr criterion will also take effect for the macro shear fractures. According to Coulomb–Mohr criterion in the effective stress form:

(1) $$\tau =C_{0}+\sigma \prime \tan\varphi ,$$

where, τ is the shear strength, $C_{0}$ and φ are the cohesion and angle of internal friction, respectively. $\sigma^{\prime }$ is the effective normal stress defined as the difference between the total normal stress and water pressure. In theory, the effective normal stress $\sigma^{\prime }$ will decrease with the water pressure $p_{w}$ *P*_w_ applied on the shear planes. In addition, the angle of internal friction φ of the shear planes will also be lowered due to the existence of water pressure. Consequently, shear strength τ will be decreased. This mechanism also contributes to the strength reduction of NRG01 granite samples under the effect of water pressure.

The different fracturing patterns dependent on the water pressure are very interesting observations in this study. With the water flowing into the pre-existing micro cracks and new cracks initiated by the triaxial loading, the granite sample can be considered as an intact specimen including randomly distributed cracks with pore water pressures. Because the samples are under triaxial compressive conditions, most of the cracks with random orientations are under compressive–shear stress states. According to the systematic LEFM based analyses on the failure mechanism of compressive–shear cracks affected by pore water pressures, Zhao et al. [23] proposed that the cracks are prone to coalesce by shearing through the rock bridges between them under higher pore water pressures. This research may provide us with an explanation to understand why shear planes are easier to be formed in the NRG01 granite samples under the effect of water pressures.

Compared with the brittle tensile failure in the samples with no water pressure, shearing through the rock bridges and the formation of macro shear planes are more prone to lead to more obvious yielding processes and gradual stress drops. This type of damage process usually refers to a gradual release of energy, and it can also be a reason for the application of water injection for mitigating the violent failure of surrounding rock mass during underground excavation.

### 3.2. Influences of Various Confinements and Water Pressures

Based on the observations and analyses on the first group of experimental studies, we have learned some effects of water pressure on the failure behavior of NRG01 granite samples. However, a more systematic group of laboratory tests should be studied for a more comprehensive understanding of the influences of confinement and water pressure. According to the test results in Section 3.1, higher water pressure may lead to more obvious influences on the failure behaviors of NRG01 granite samples. However, the water pressure cannot be higher than the confinement for ensuring the heat-shrink tubing working in a good condition. Considering the limited number of samples and consistency of the tests, water pressure is set as 0.8 times the corresponding confinement for each of the tests, aimed at obtaining some characteristics as clearly as possible. Table 2 shows the confinements and water pressures designed for this group of experiments.

**Figure 6:**
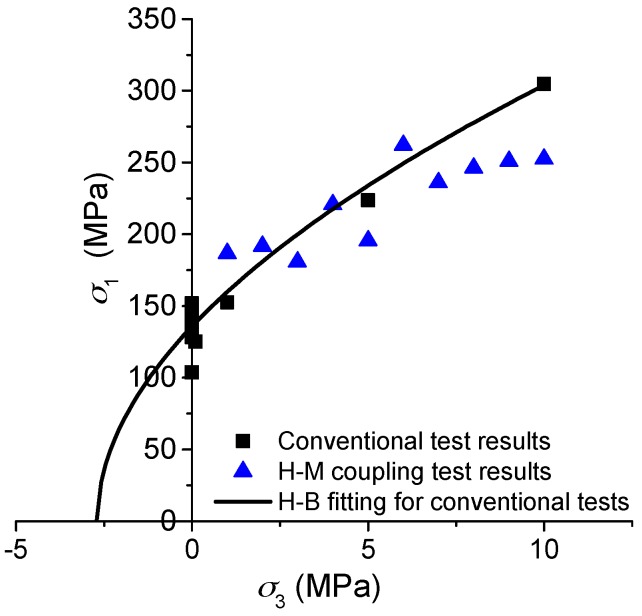
Peak strength values of NRG01 granite samples affected by water pressure (blue triangles) compared with conventional test results under different confinements without any water pressure (black squares).

Figure 6 presents the peak strength values obtained from the hydro-mechanical (H-M) coupling tests and the conventional compressive tests under different confinements with no water pressures. For tests under higher confinements and water pressure (*σ*_3_ > 4.0 MPa and *p*_w_ > 3.2 MPa), the strength values are generally lower than the Hoek–Brown fitting curve for the conventional test results. However, for tests under lower confinements and water pressure (*σ*_3_ ≤ 4.0 MPa and *p*_w_ ≤ 3.2 MPa), the strength values are almost around the fitted curve. It appears that the water pressure has more obvious influence on the peak strength under higher confinements and water pressures. The main reason should be that the water pressures *p*_w_ ≤ 3.2 MPa are not high enough to reduce the fracture toughness of the pre-existing micro-cracks significantly, as explained in Section 3.1.

**Figure 7:**
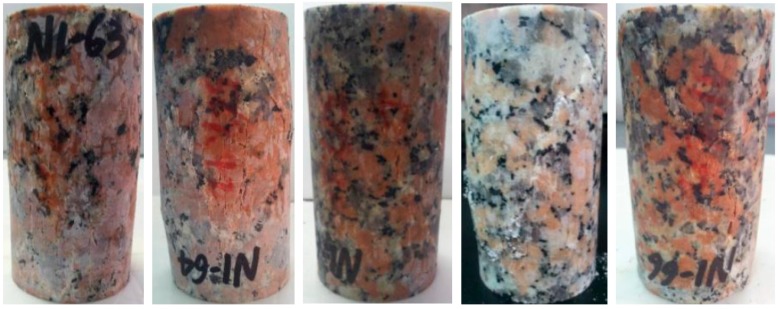

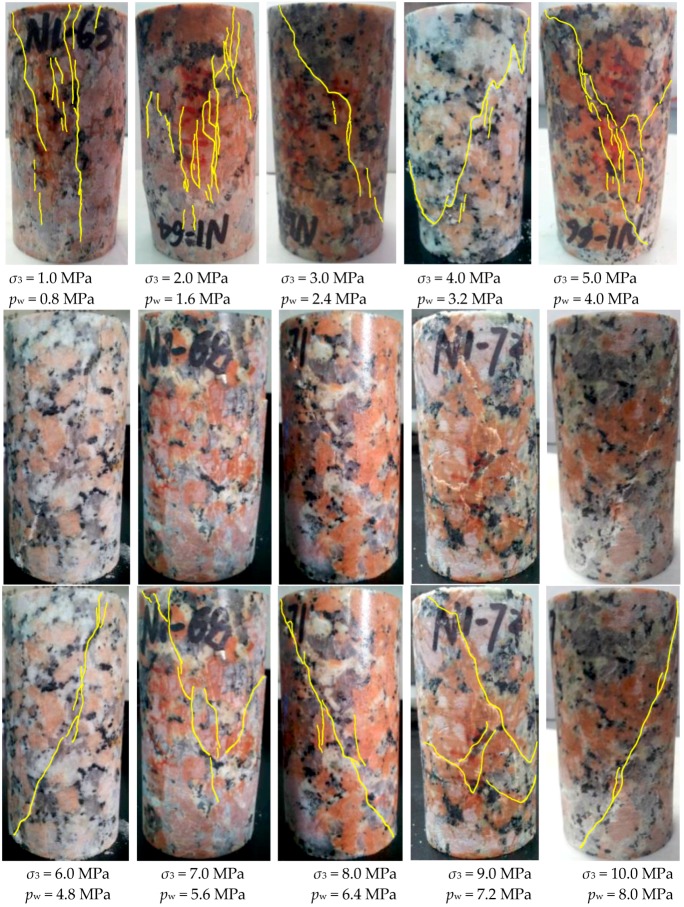
Fracturing patterns of NRG01 granite samples under various confinements and water pressure. The yellow lines in the lower pictures show the fractures compared with the original pictures of the samples.

Based on fracturing patterns presented in Figure 7, it is found that through shear planes are formed in almost all the samples for the cases of *σ*_3_ ≥ 4.0 MPa and *p*_w_ ≥ 3.2 MPa. However, the failure patterns are dominated by vertical tensile fractures for the cases of *σ*_3_ < 3.0 MPa and *p*_w_ < 2.4 MPa, while the case of *σ*_3_ = 3.0 MPa and *p*_w_ = 2.4 MPa exhibits a transitionary condition with mixed tensile and shear fractures.

It is well known that rock failure is always dominated by tensile fractures under lower confinement, and it changes to be dominated by shear fractures under higher confinement. For NRG01 granite samples, the conventional compressive tests show that tensile fractures dominate the failure mode under the confinement *σ*_3_ ≤ 10 MPa. The vertical tensile fractures can be observed clearly in the samples under the confinements *σ*_3_ = 5 and 10 MPa with no effect of water pressure (Figure 5).

However, based on the fracturing patterns observed in Figure 7, with the effect of water pressure, the shear fractures are formed and dominate the failure patterns under much lower confinements (*σ*_3_ = 4.0 MPa). These test results also present more evidence for the observations in Section 3.1, that it is much easier to form shear fracturing patterns for the NRG01 granite samples under the effect of water pressure. 

Based on the observations and analyses in this section, it is found that water pressure plays a significant role in the failure behaviors of NRG01 granite samples, including peak strength, fracturing pattern, and stress–strain response under different confinements. These failure behaviors are of great importance for the stabilization of underground repositories. In addition, they may also have influences on the seepage characteristics in the surrounding rock, which will be studied in the next section.

## 4. Seepage Characteristics of NRG01 Granite Samples Dependent on the Failure Behaviors Affected by Confinement and Water Pressure

### 4.1. Stages and Characteristics of the Seepage Process

In this study, inflow rate is analyzed to assess the seepage behavior of NRG01 granite samples during the damage and fracturing process for tests under the effects of confinement and water pressure. Inflow rate *Q* (m^3^/s) is defined as the rate of water volume flowing into the samples. Unlike permeability tensor used in many studies, the inflow rate *Q* can be applied as a scalar parameter to quantitatively characterize the evolutionary seepage behavior of the rock during the damage and fracturing process. It can be measured continuously during the whole test process, instead of interrupting the test and remaining a period at some pre-designed key points.

**Figure 8:**
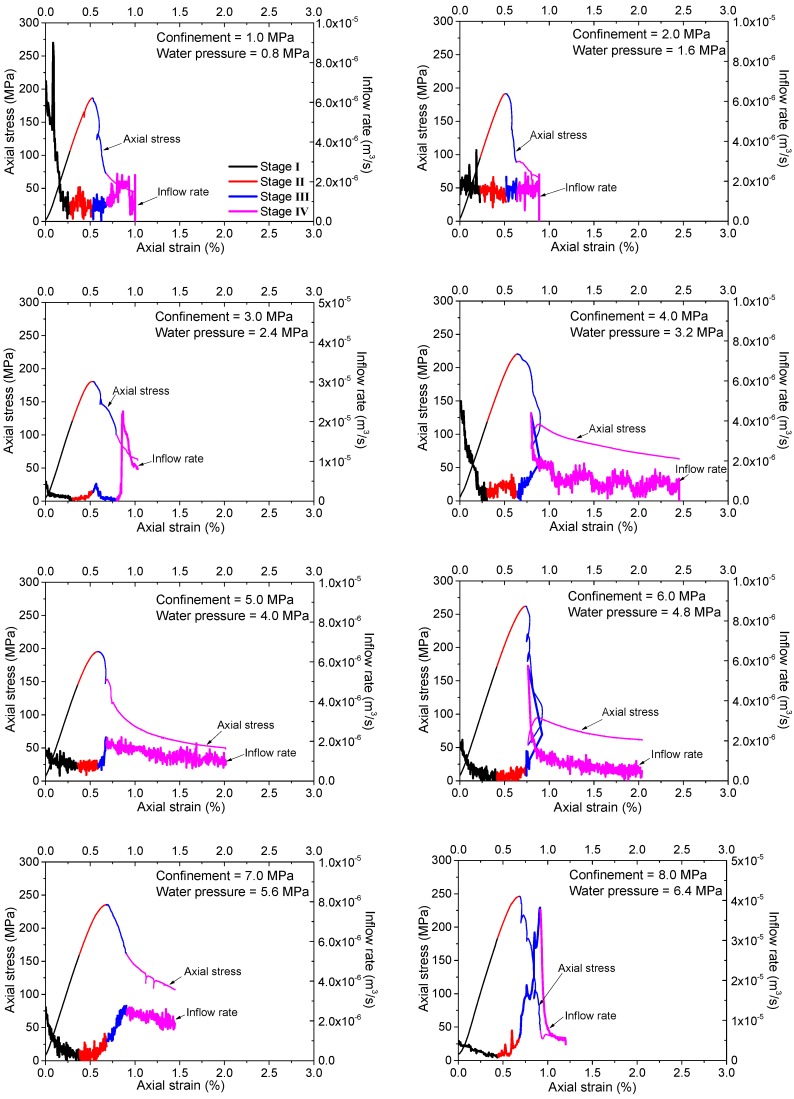

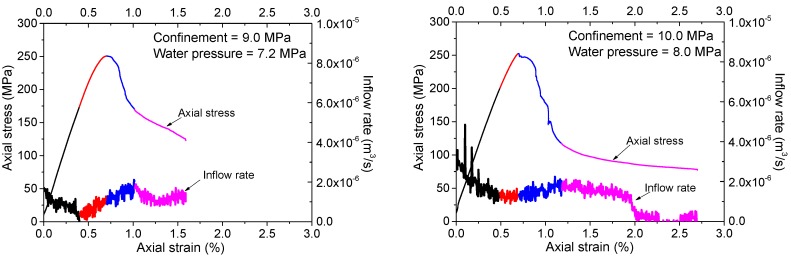
Axial stress–strain and inflow rate curves for the tests under different confinements and water pressures. The black, red, blue and magenta colors refer to stage I, II, III and IV, respectively.

The changing inflow rate curve is plotted comparing with the axial stress–strain curve for each of the tests (Figure 8). It should be noted that different samples show quite different initial inflow rates, which are resulted from the scattered distributions of the pre-existing micro-cracks in the unsaturated samples. It is believed that this difference can be ignored, as only the evolutionary characteristics of the inflow rates are of interest in this study. Generally speaking, each of the inflow rate curves can be divided into four stages with the increasing loading and the evolution of damage and fracturing. Nonetheless, different seepage behaviors can be observed according to the comparison of the inflow rate curves under different confinements and water pressures.

(1) For the tests under higher confinements and water pressures (*σ*_3_ ≥ 4.0 MPa and *p*_w_ ≥ 3.2 MPa), the general characteristics of various stages of the inflow rate curves can be observed as follows:
  - Stage I: Inflow rate decreases quickly during the initial period of axial loading (black portions of the curves in Figure 8).
  - Stage II: Inflow rate increases slightly until the reach of peak strength (red portions of the curves in Figure 8).
  - Stage III: Inflow rate increases significantly after the peak strength (blue portions of the curves in Figure 8).
  - Stage IV: Inflow rate decreases until the end of the test (magenta portions of the curves in Figure 8).
(2) For the tests under lower confinements and water pressures (*σ*_3_ < 4.0 MPa and *p*_w_ < 3.2 MPa), the inflow rate curves show some different features as follows (for the last three stages, the transitionary case of *σ*_3_ = 3.0 MPa and *p*_w_ = 2.4 MPa shows some complex characteristics and will be described specially):
  - Stage I: Similarly, the inflow rate decreases during the initial period of axial loading.
  - Stage II: For the cases of *σ*_3_ < 3.0 MPa and *p*_w_ < 2.4 MPa, there are some vibrations in the inflow rate curves, but no general increase or decrease is observed at this stage; For the case of *σ*_3_ = 3.0 MPa and *p*_w_ = 2.4 MPa, there is a slight increase in inflow rate, which turns to be similar to the observations for the tests under higher confinements and water pressures.
  - Stage III: For the cases of *σ*_3_ < 3.0 MPa and *p*_w_ < 2.4 MPa, the inflow rate is almost constant at this stage. For the case of *σ*_3_ = 3.0 MPa and *p*_w_ = 2.4 MPa, the inflow rate increases for a very short term after the peak strength and then decreases immediately.
  - Stage IV: For the cases of *σ*_3_ < 3.0 MPa and *p*_w_ < 2.4 MPa, the inflow rate shows some vibrations or keeps almost constant in this stage. For the case of *σ*_3_ = 3.0 MPa and *p*_w_ = 2.4 MPa, the inflow rate increases significantly to a very high value and then decreases gradually.

### 4.2. Discussion

What is the reason for the changing inflow rate? What causes the differences in various stages of the tests under different confinements and water pressures? Again, as discussed before, NRG01 granite samples are not sensitive to the moisture content, therefore the failure behaviors, especially changing fracturing patterns induced by various confinements and water pressures should be the key factors for the varying of inflow rate.

All the cases show a similar behavior of inflow rate (decreasing) in stage I. According to the curves presented in Figure 8, stage I corresponds to the initial process of axial loading, almost including the crack closure and linear elastic loading periods. In this stage, the pre-existing micro cracks supply paths for water inflow with relatively high inflowing rate. However, as some pre-existing cracks start to close under increasing loading, and the open cracks are gradually filled with water while very few new cracks have been initiated, the inflow rate turns out to be decreased.

Stage II almost corresponds to the stable or unstable crack growth period in the pre-peak region. In this stage, new cracks begin to initiate and propagate and some crack coalescences occur, which may supply new paths for water seepage. That is why a slight, but nonetheless obvious, increase in inflow rate can be observed for cases of higher confinements and water pressures (*σ*_3_ ≥ 4.0 MPa and *p*_w_ ≥ 3.2 MPa). However, based on the fracturing patterns shown in Figure 7 and the analyses in the last section, for the case of lower confinements and water pressures (*σ*_3_ < 3.0 MPa and *p*_w_ < 2.4 MPa), the crack coalescences are prone to form macro tensile fractures. These tensile fractures are more dispersedly distributed and usually have relatively higher roughness. In addition, they are more easily closed under confinement so they usually have lower apertures., Therefore, some vibrations of inflow rate may occur but no general increase is observed. The transitionary case of *σ*_3_ = 3.0 MPa and *p*_w_ = 2.4 MPa has a complex failure pattern combined with the shear and tensile fractures. The shear part connects the upper sample end where water flows into the granite, so the curve shows an increase of inflow rate like the cases with higher confinements and water pressures at this stage.

Stage III corresponds to the initial part of the post-peak period when the failure of the sample has occurred with the formation of connective macro fractures. Quite different seepage behaviors are observed in this stage for different cases owing to the formation of different macro fracturing patterns. For cases of higher confinements and water pressures (*σ*_3_ ≥ 4.0 MPa and *p*_w_ ≥ 3.2 MPa), the macro fractures are almost shear type, and the dilation induced by the slippage of the shear planes usually results in relatively lower roughness and higher apertures, which can supply better water paths with higher conductivities. Hence the inflow rate increases significantly at this stage. However, no obvious increase is observed for the cases of *σ*_3_ < 3.0 MPa and *p*_w_ < 2.4 MPa owing to the tensile fractures with higher roughness and lower apertures. For the transitionary case of *σ*_3_ = 3.0 MPa and *p*_w_ = 2.4 MPa with mixed tensile and shear fractures, the inflow rate increases for a short period owing to the formation of the shear portion of the fractures. Then, the inflow rate decreases abruptly because the shear fracture does not go through the specimen and the slippage is inhibited, while the tensile fractures cannot supply the water paths to induce an obvious increase of inflow rate.

Stage IV corresponds to the following or residual portion of the post-peak period. The decrease of inflow rate for the cases of higher confinements and water pressures (*σ*_3_ ≥ 4.0 MPa and *p*_w_ ≥ 3.2 MPa) should mainly be owing to the closing effect of the fractures during the lasting shear deformation. While for the case of *σ*_3_ < 3.0 MPa and *p*_w_ < 2.4 MPa, the durative axial loading may supply continuous tensile stresses, and some parts of the tensile fractures may be open at this stage. This may result in some temporary increase or vibrations of the inflow rate. For the transitionary case of *σ*_3_ = 3.0 MPa and *p*_w_ = 2.4 MPa with mixed tensile and shear fractures, the observed significant increase in inflow rate could be explained by the opening of the tensile fracture and the induced slippage of the shear fracture based on the observed fractures in Figure 5. However, this is just a transient event during the durative axial loading and the inflow rate decreases quickly again.

The observations and analyses based on the laboratory experiments demonstrate that the seepage behaviors are closely related to the failure behavior, especially the fracturing patterns of NRG01 granite, which are influenced by the confinements and water pressures. These findings may provide us with a better understanding of the different seepage characteristics in the different parts of the surrounding rock suffering from different stress conditions and water pressures, like the examples given in [38,39]. Accordingly, the permeability tensor should also be affected by the failure behavior dependent on the confinements and water pressures. This will be researched in future studies with the improvement of the experimental devices.

## 5. Conclusions

Based on the observations and analyses on the failure and seepage behaviors of NRG01 granite samples through a series of laboratory experiments, it is found that the confinement and water pressure have significant influences on the failure properties of NRG01 granite samples, including strength, fracturing patterns and stress–strain response. These failure behaviors, in turn, affect the evolutionary seepage characteristics during the damage process. This work is helpful for understanding the mechanism of the varying seepage features under different confinements and water pressures.

There are several main findings as follows:

(1) Strength reduction properties of NRG01 granite samples are investigated under the effects of confinement and water pressure. In addition, the complete axial stress–strain curves show more obvious yielding process in the pre-peak region and the stress drops in a more gradual manner as a result of water pressure under different confinements. These failure behaviors are of importance for the stability of the host rock.
(2) Shear planes are more prone to be formed for NRG01 granite samples with the effect of water pressure, even under much lower confinements than the predictions by conventional triaxial compressive test results. In this study, the failure patterns are still dominated by tensile fracturing under confinement of 10.0 MPa with no water pressure. However, shear fracturing dominated failure pattern is formed even for the test under confinement of 4.0 MPa with water pressure of 3.2 MPa. The influences on the fracturing patterns are paramount for both rock stability and the seepage behaviors.
(3) The water inflow rate curves are divided into four stages, showing different seepage behaviors dependent on the failure behaviors influenced by the confinement and water pressure. The shear fractures induced by higher confinement and water pressure (*σ*_3_ ≥ 4.0 MPa and *p*_w_ ≥ 3.2 MPa) result in various degrees of inflow rate increase during the process of crack growth and fracture formation. This is followed by a decrease with the closing of the fractures in the last stage. While tensile fractures induced by lower confinement and water pressure (*σ*_3_ < 3.0 MPa and *p*_w_ < 2.4 MPa) do not show such obvious changes of inflow rate in the corresponding stages, and the transitionary condition (*σ*_3_ = 3.0 MPa and *p*_w_ = 2.4 MPa) with mixed shear and tensile fractures leads to complex seepage behaviors.

These findings may have several contributions as follows: (1) A more comprehensive understanding on the failure and seepage mechanism of NRG01 granite is supplied based on the observed failure behaviors and the analyses on the seepage behaviors affected by the failure behaviors; (2) These failure and seepage mechanism of NRG01 granite are useful for the design and construction of the HLW repository, considering both mechanical stability and prevention of radionuclide transportation. In addition, the research method can also be applied in other rock engineering with different types of surrounding rock mass; (3) These observed failure patterns in the laboratory experiments provide more evidences for calibration of numerical models considering the effects of confinement and water pressure. In the future, a larger range of confinement and water pressure will be considered in more systematic studies. In addition, the permeability tensor will be investigated in such a framework, and the effect of temperature will also be considered in the failure and seepage behaviors of NRG01 granite samples.

## Figures and Tables

**Table 1:** Design for the tests under fixed confinements but altering water pressures.

Sample No.	Height (mm)	Diameter (mm)	Density (g/cm^3^)	Confining Pressure (MPa)	Water Pressure (MPa)
N1-14	100.13	49.51	2.65	5.0	0
N1-66	100.06	49.61	2.66	5.0	4.0
N1-29	99.67	49.99	2.63	10.0	0
N1-19	100.13	49.57	2.65	10.0	6.0
N1-67	100.09	49.02	2.65	10.0	8.0

**Table 2:** Design for the tests under different confinements and water pressures.

Sample NO.	Height (mm)	Diameter (mm)	Density (g/cm^3^)	Confining Pressure (MPa)	Water Pressure (MPa)
N1-63	100.02	49.61	2.65	1.0	0.8
N1-64	100.04	49.54	2.64	2.0	1.6
N1-65	100.09	49.56	2.66	3.0	2.4
N1-69	100.15	49.55	2.64	4.0	3.2
N1-66	100.06	49.61	2.66	5.0	4.0
N1-70	100.15	49.63	2.65	6.0	4.8
N1-68	100.10	49.67	2.62	7.0	5.6
N1-71	100.21	49.62	2.66	8.0	6.4
N1-72	100.06	49.58	2.66	9.0	7.2
N1-67	100.09	49.02	2.65	10.0	8.0
